# Supplementary material for: Catalytic production of low-carbon footprint sustainable natural gas
Source: Nat Commun. 2022 Jan 11;13:258. doi: 10.1038/s41467-021-27919-9 (PMC8752773; doi:10.1038/s41467-021-27919-9)
Supplement: Supplementary file 1 — Supplementary Information [file 41467_2021_27919_MOESM1_ESM.pdf]

Supplementary Information

Catalytic Production of Low-carbon Footprint Sustainable Natural Gas

Si et al.

This PDF file includes:

Supplementary Methods
Supplementary Figures 1 to 15
Supplementary Tables 1 to 10
Supplementary References

Supplementary Methods

The fractionation of organosolv lignin

Organosolv lignin was separated according to the previous publication[1]. Briefly, 30 g of beech sawdust and 360 mL of ethanol and water mixed solvent (1/1, v/v) were introduced into a 500 mL autoclave with a mechanical stirrer. The reactor was sealed and then heated to 170 ℃ within 25 min. The reactor was held at 170 ℃ for 200 min with a stirring at 500 rpm. After the reaction, the autoclave was rapidly cooled down to the room temperature in the ice-water bath. The solid and liquid fractions were separated with vacuum filtration, and the separated solid was washed with 360 mL of ethanol and water mixed solvent (1/1, v/v). The combined filtrate and washing solutions were added into 1500 mL of water under stirring at 500 rpm for 2 h. Organosolv lignin was precipitated from the solution and separated with vacuum filtration, which was washed with water and dried at 40 ℃ for 48 h in the vacuum oven.

Supplementary Figures

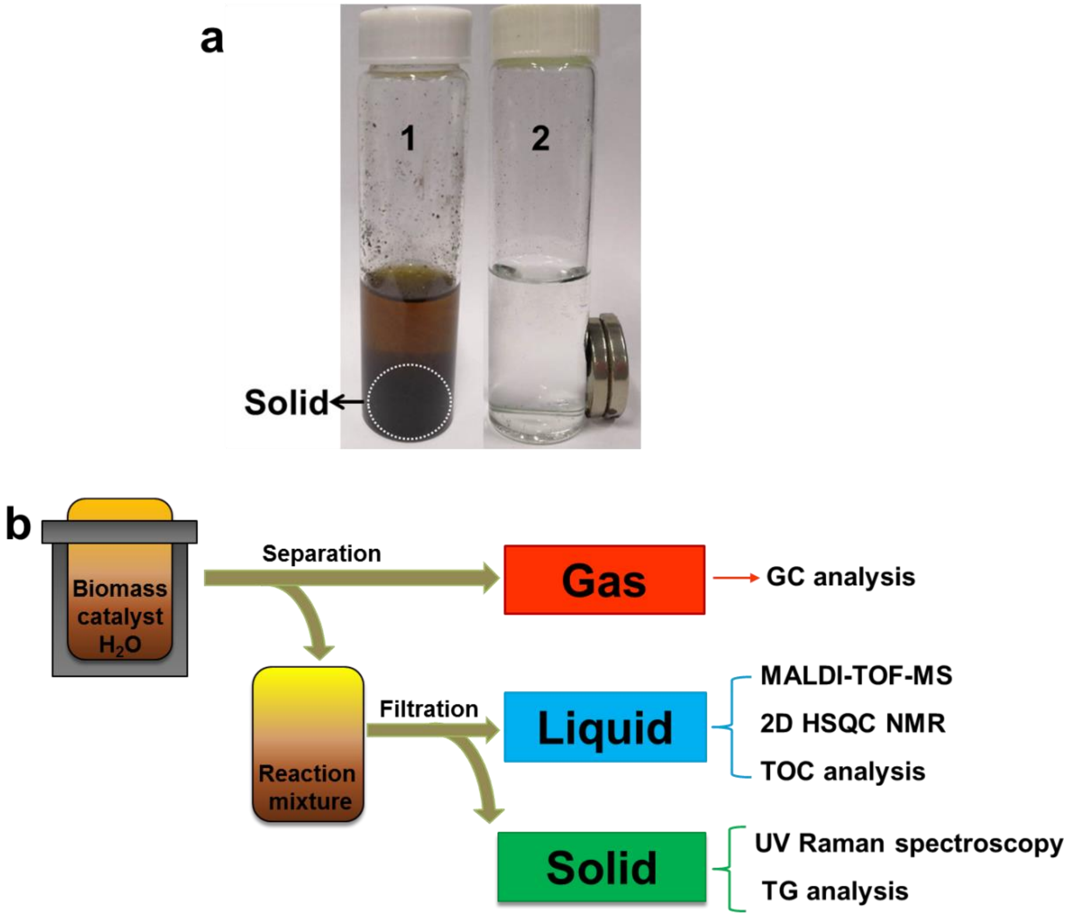

Supplementary Fig. 1 Catalytic conversion of beech sawdust to bio-natural gas. a Images of beech sawdust in water after the reaction (1-without catalyst, 2-with the nickel-based alloy catalyst). **b** Gas, liquid and solid fractions were collected and analyzed separately after the reaction. Reaction condition: 1.0 g beech sawdust, 5.2 mmol/0.38 g nickel-based alloy catalyst, 20 mL H_2O, 4 MPa H_2, 250 ℃, 5 h.

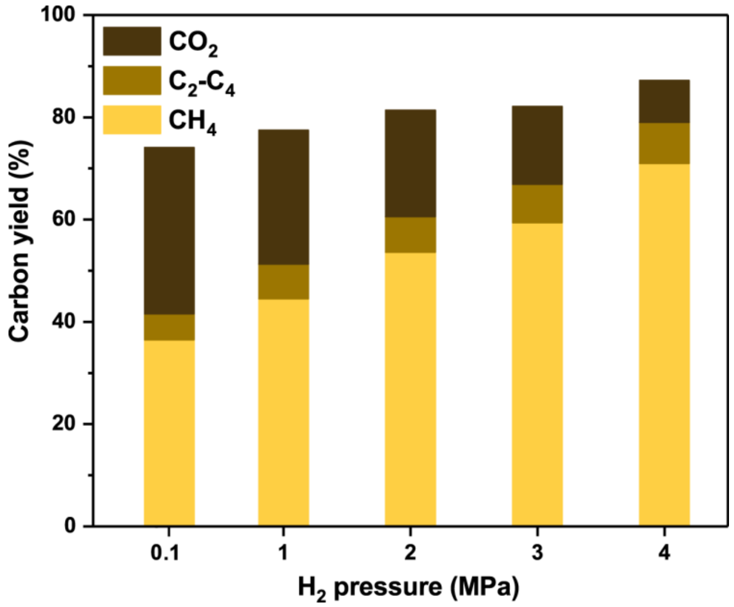

Supplementary Fig. 2 Carbon yield of gas products with the different H_2 pressure. Reaction condition: 1.0 g beech sawdust, 5.2 mmol nickel-based alloy catalyst, 20 mL H_2O, 300 ℃, 5 h.

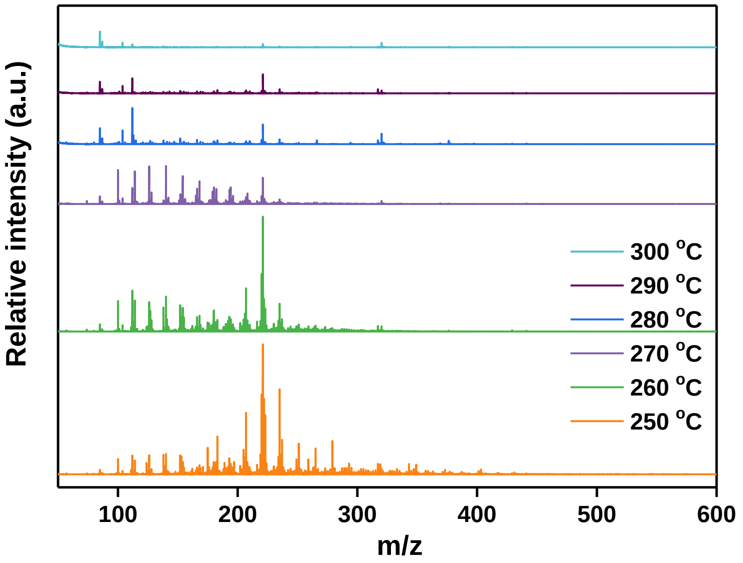

Supplementary Fig. 3 MALDI-TOF-MS analysis of liquid products at various reaction temperatures. Reaction condition: 1.0 g beech sawdust, 5.2 mmol nickel-based alloy catalyst, 20 mL H_2O, 4 MPa H_2, 5 h.

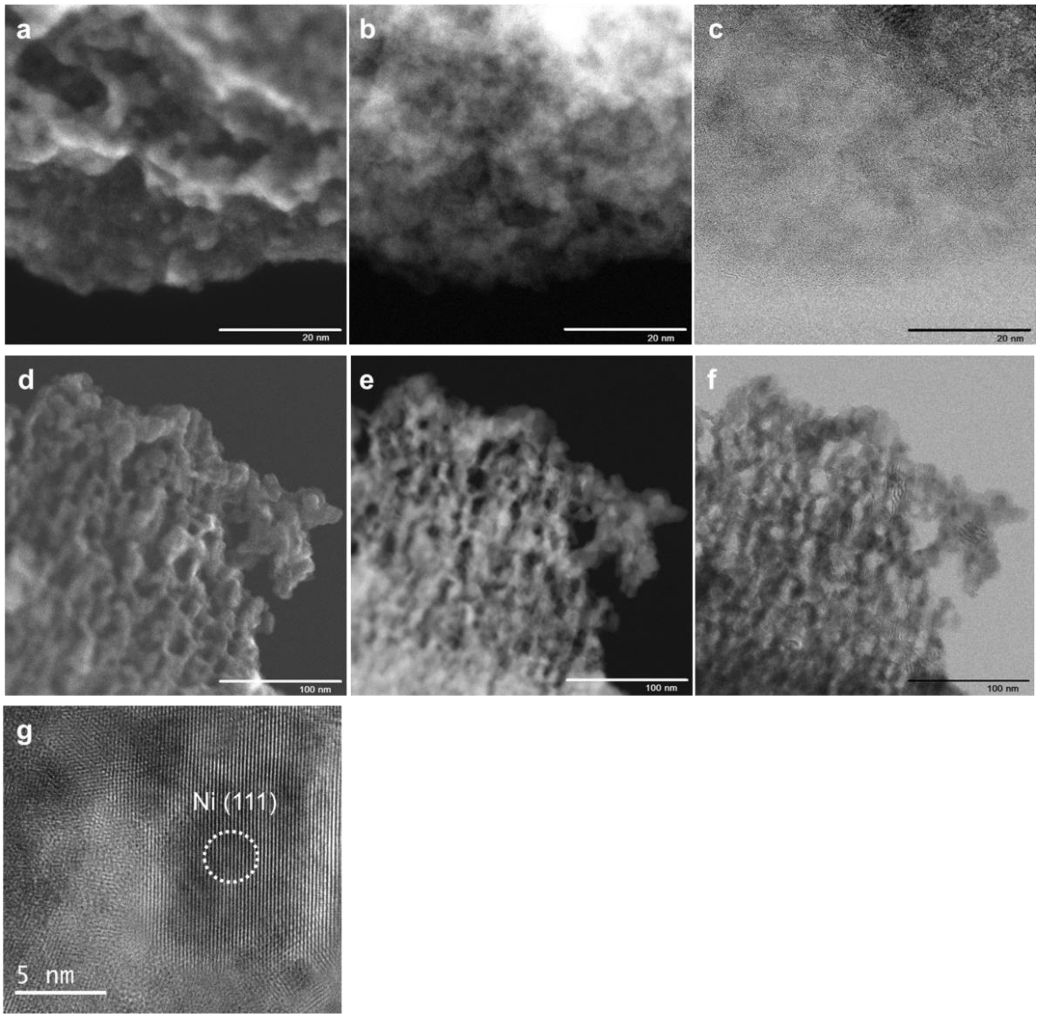

Supplementary Fig. 4 STEM images. a-c The nickel-based alloy catalyst. **d-g** Commercial nickel catalyst.

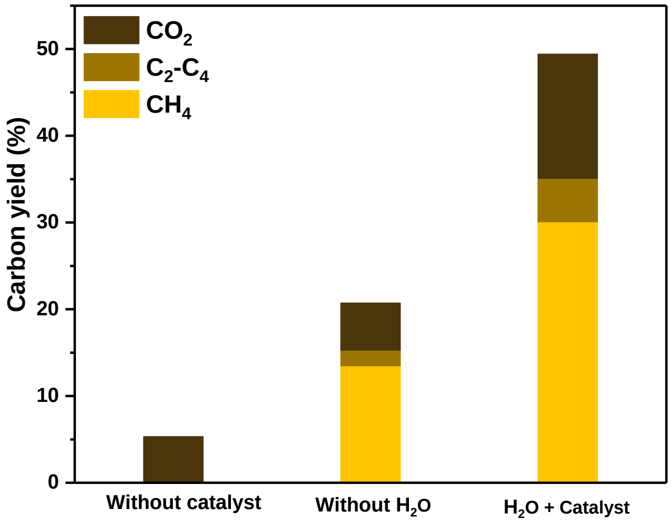

Supplementary Fig. 5 Effects of H_2O and catalyst on the catalytic conversion of raw biomass to bio-natural gas. The reactions were performed under the same operating conditions without catalyst or H_2O.

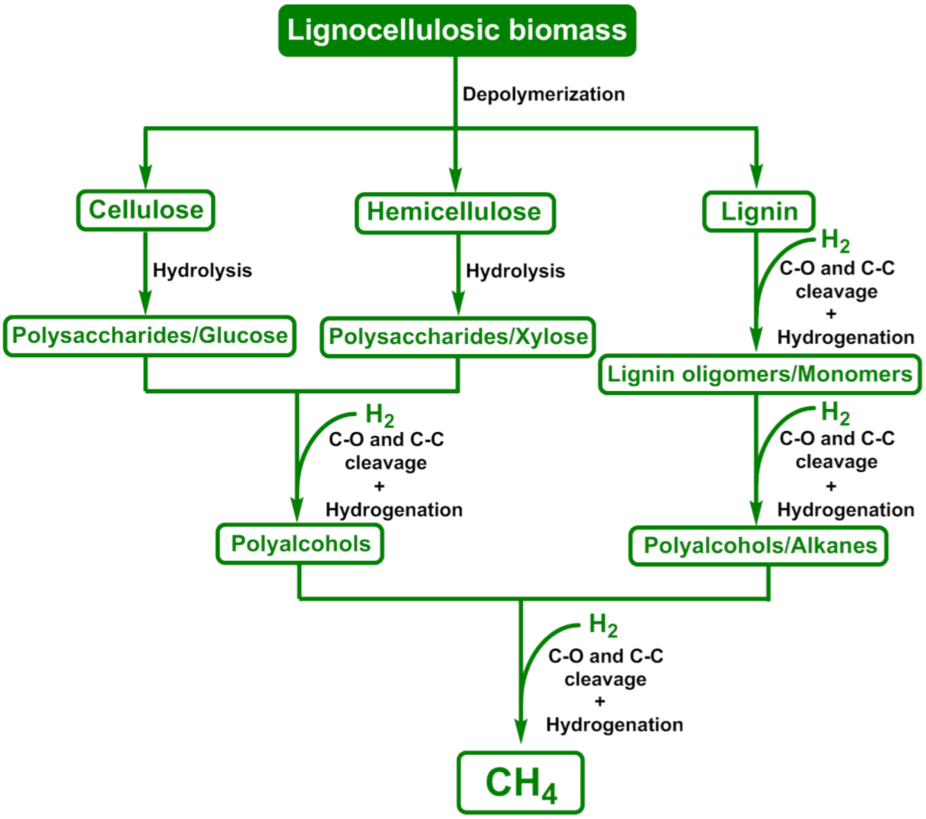

Supplementary Fig. 6 The process of catalytic lignocellulosic biomass to natural gas. It mainly included the depolymerization of lignocellulosic biomass and the sequential conversion of biomass constituents to CH_4.

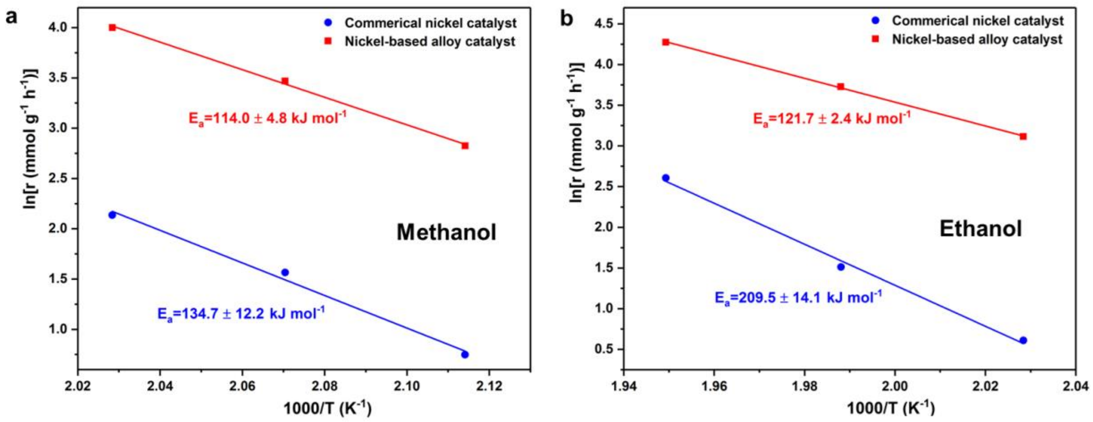

Supplementary Fig. 7 Kinetic measurements of methanol and ethanol conversion.

Arrhenius plots for **a** methanol, **b** ethanol over nickel based catalysts.

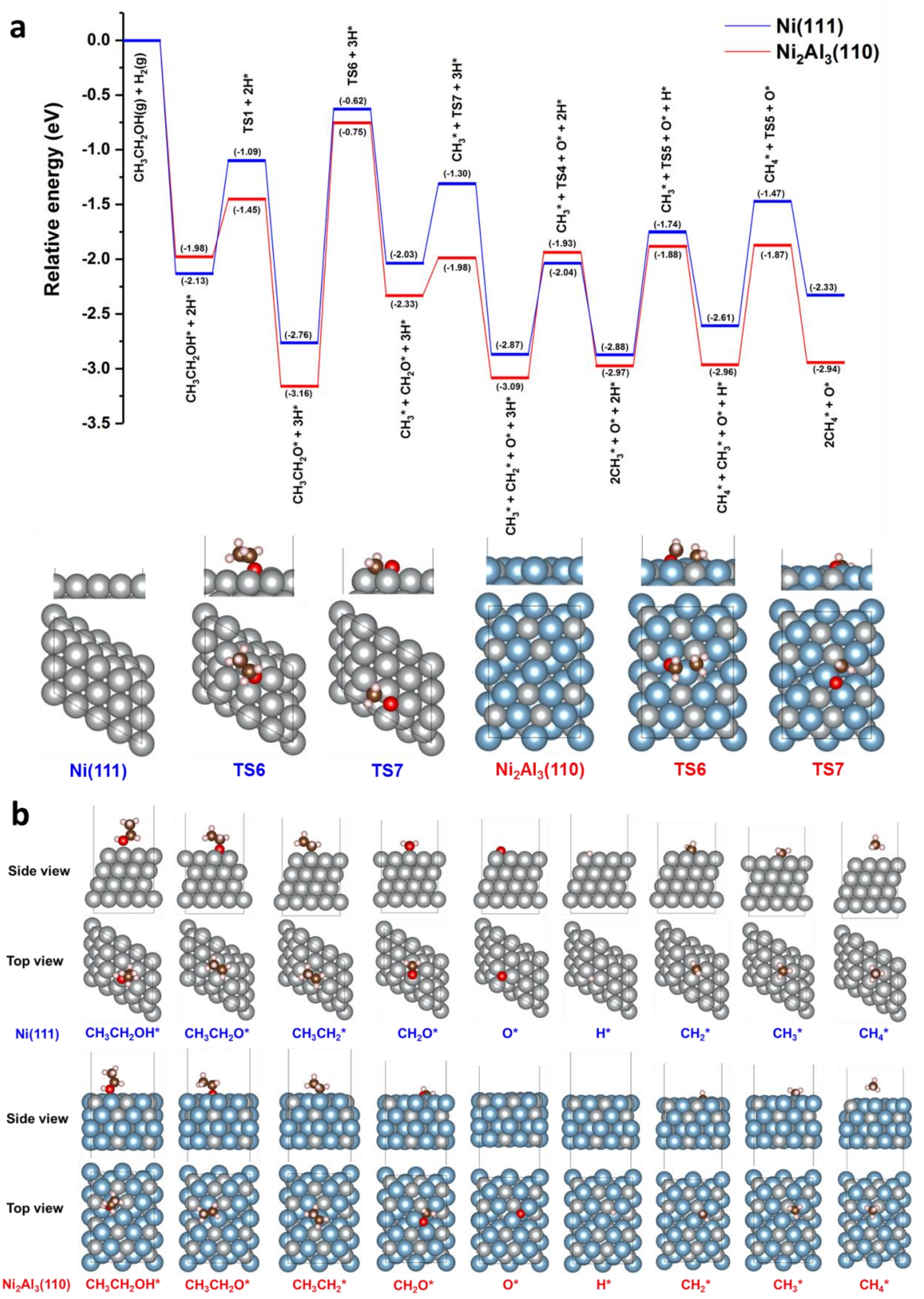

Supplementary Fig. 8 Reaction pathways of C-C bond preferentially cleaved in ethanol compound. a Energy profiles for CH_3CH_2OH dissociation on Ni(111) and Ni_2Al_3(110) surfaces of C-C bond preferentially cleaved. The *x* axis shows the

reaction intermediates, the *y* axis shows the relative energy of each state. **b** The side and top view of reaction species on Ni(111) and Ni_2Al_3(110).

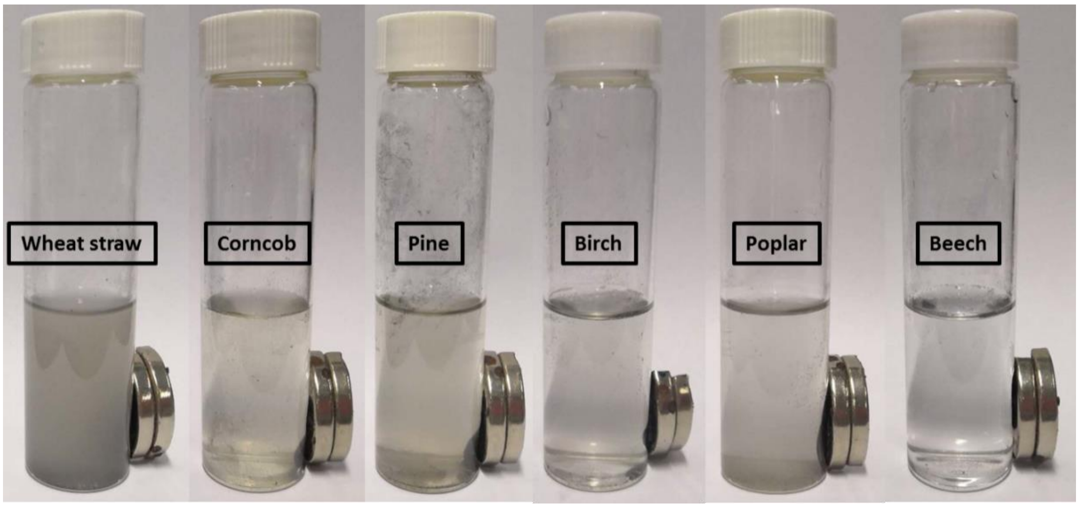

Supplementary Fig. 9 Catalytic conversion of agricultural and forestry residues. Images of agricultural and forestry residues in water after the reaction over the nickel-based alloy catalyst. Reaction condition: 1.0 g substrate, 5.2 mmol nickel-based alloy catalyst, 20 mL H_2O, 4 MPa H_2, 300 ℃, 5 h.

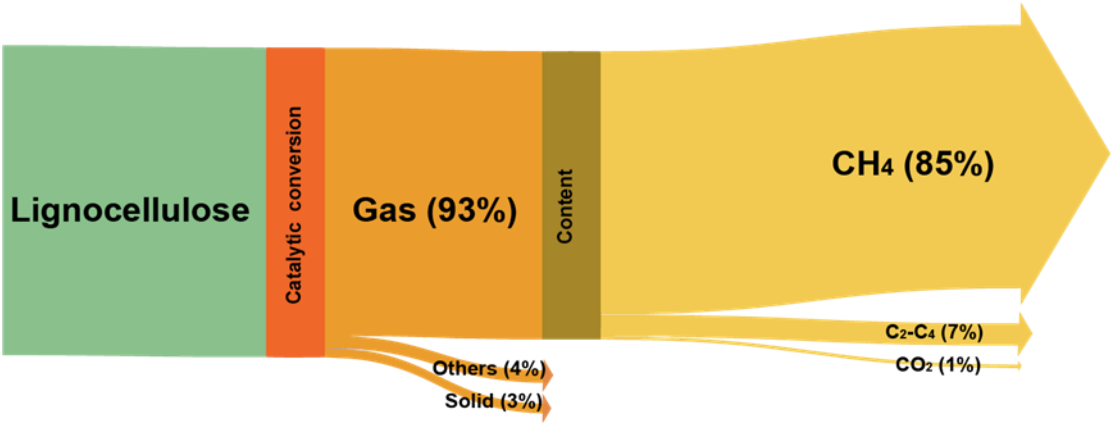

Supplementary Fig. 10 Sankey diagram based on carbon balance in beech sawdust transformation process. Reaction condition: 0.5 g beech sawdust, 2.6 mmol nickel-based alloy catalyst, 20 mL H_2O, 4 MPa H_2, 300 ℃, 5 h.

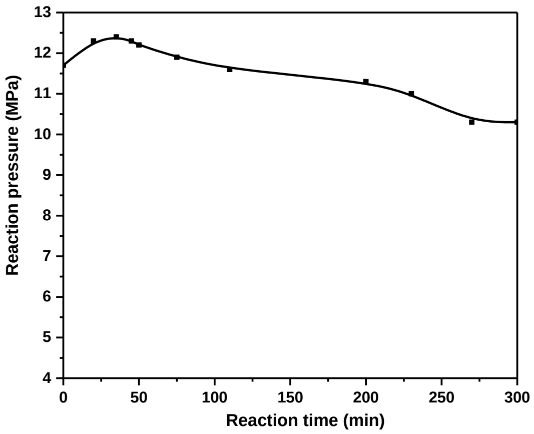

Supplementary Fig. 11 The reaction pressure during the reaction. Reaction condition: 1.0 g beech sawdust, 5.2 mmol nickel-based alloy catalyst, 20 mL H_2O, 4 MPa H_2, 300 ℃, 5 h.

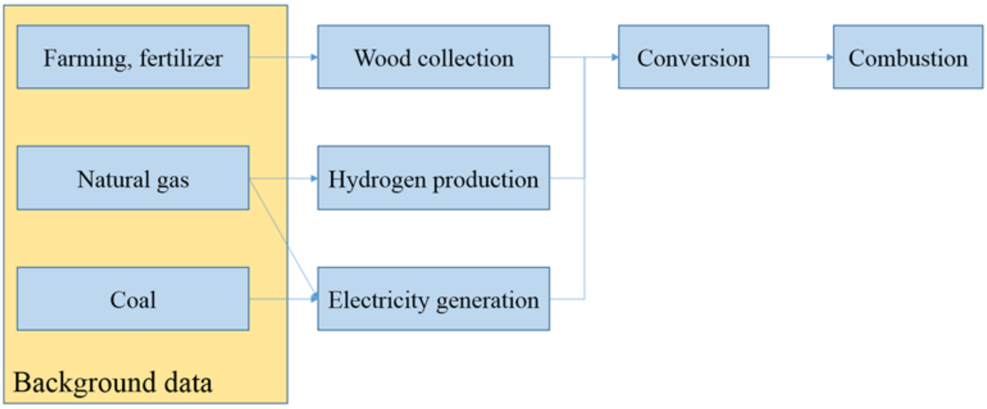

Supplementary Fig. 12 System boundary of bio-natural gas. It contains conversion and combustion processes, feedstocks, required materials and utilities.

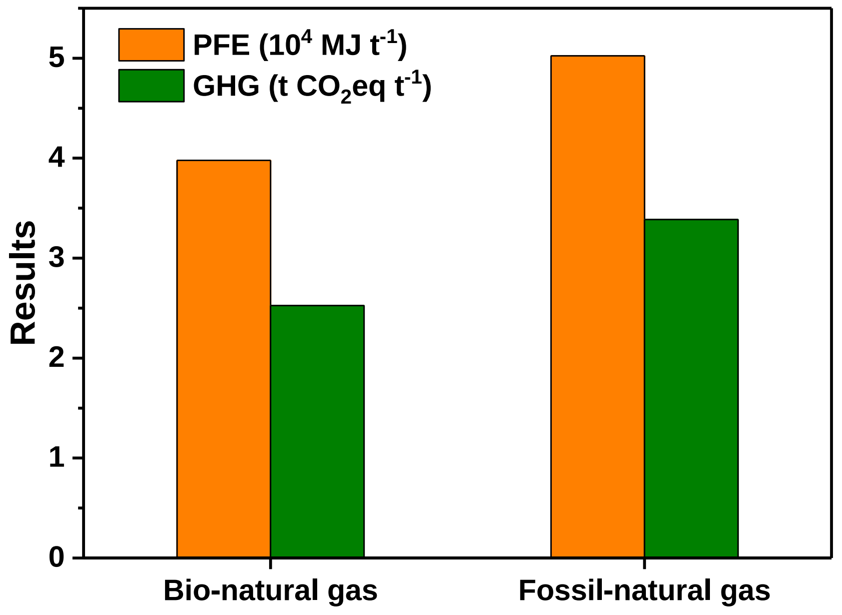

Supplementary Fig. 13 Performance of bio-natural gas and fossil-natural gas.

The PFE and GHG for the fossil- and bio-natural gas.

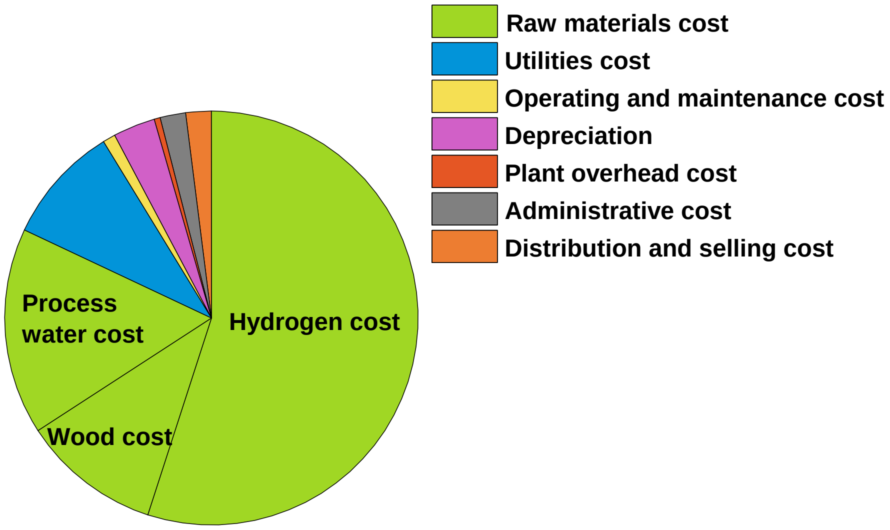

Supplementary Fig. 14 The detail breakdown of TPC of bio-natural gas. It is mainly comprised of hydrogen cost, wood cost, process water cost, utilities cost, etc.

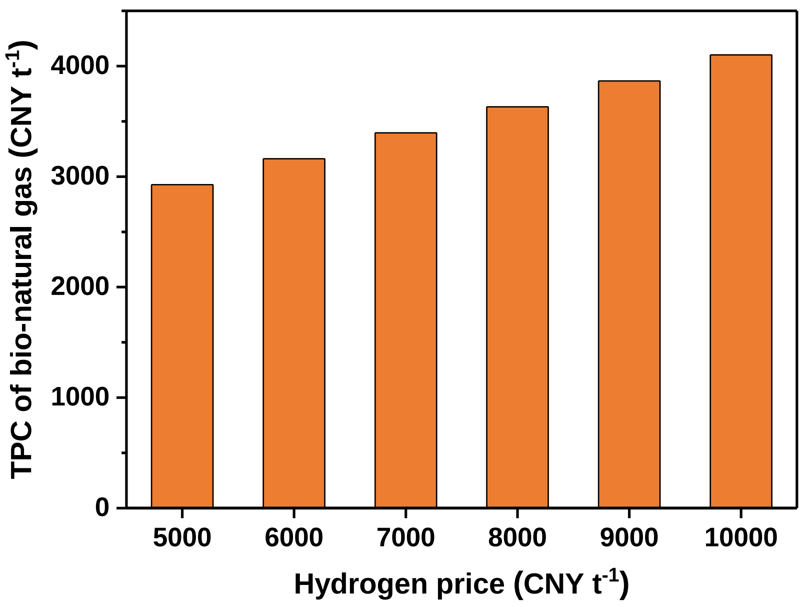

Supplementary Fig. 15 Effects of hydrogen price on TPC of bio-natural gas. The range of hydrogen price is 5000-10000 CNY t^{-1}.

Supplementary Tables

Supplementary Table 1 Carbon yield and product distribution of CH_4, C2-C4 hydrocarbons and CO_2 after the reaction at various temperatures

Entry*	Temp. (℃)	Carbon yield (%)						Product distribution (mol%)		
		CH_4	C_2H_6	C_3H_8	C_4H_{10}	CO_2	Total	CH_4	C_2-C_4	CO_2
1	250	50.9	3.7	1.5	0.5	2.4	59.0	91.3	4.4	4.3
2	260	54.6	3.9	1.8	0.7	3.0	64.0	90.5	4.5	5.0
3	270	57.3	3.9	2.3	0.9	5.2	69.6	87.5	4.5	8.0
4	280	65.0	4.0	2.1	0.9	7.5	79.5	86.1	3.9	10.0
5	290	69.9	4.0	2.2	0.9	7.5	84.5	86.9	3.7	9.4
6	300	71.0	4.1	2.7	1.2	8.2	87.2	86.1	4.0	9.9
7	270†	24.7	1.4	1.7	1.2	4.3	33.3	80.9	5.2	13.9

*The carbon molar yield of gas products was quantified by GC analysis, reaction condition: 1.0 g beech sawdust, 5.2 mmol nickel-based alloy catalyst, 20 mL H_2O, 4 MPa H_2, 5 h.

†Reaction condition: 1.0 g beech sawdust, 6.2 mmol commercial nickel catalyst, 20 mL H_2O, 4 MPa H_2, 5 h.

Supplementary Table 2 Carbon yield and product distribution of CH_4, C2-C4 hydrocarbons and CO_2 after the reaction with different solvents

Entry	Reaction condition	Carbon yield (%)						Product distribution (mol%)		
		CH_4	C_2H_6	C_3H_8	C_4H_{10}	CO_2	Total	CH_4	C_2-C_4	CO_2
1	Without catalyst*	0.1	0.0	0.0	0.0	5.2	5.3	1.9	0.0	98.1
2	Without H_2O†	13.5	1.4	0.4	0.0	5.4	20.7	65.2	8.7	26.1
3	H_2O and catalyst‡	30.1	3.2	1.4	0.4	14.3	49.4	60.9	10.1	29.0

*Reaction condition: 1.0 g beech sawdust, 20 mL H_2O, 2 MPa H_2, 250 ℃, 2 h.

†1.0 g beech sawdust, 5.2 mmol nickel-based alloy catalyst, 20 mL n-tetradecane, 2 MPa H_2, 250 ℃, 2 h.

‡1.0 g beech sawdust, 5.2 mmol nickel-based alloy catalyst, 20 mL H_2O, 2 MPa H_2, 250 ℃, 2 h.

Supplementary Table 3 Carbon yield and product distribution of CH_4, C2-C4 hydrocarbons and CO_2 after the reaction with different substrates

Entry*	Substrate	Carbon yield (%)						Product distribution (mol%)		
		CH_4	C_2H_6	C_3H_8	C_4H_{10}	CO_2	Total	CH_4	C_2-C_4	CO_2
1	Glucose	87.3	4.6	1.5	0.8	0.2	94.5	96.5	3.3	0.2
2	Xylose	85.2	4.5	0.9	0.4	0.0	91.1	97.0	3.0	0.0
3	4-Propyl guaiacol†	57.0	0.4	1.1	1.3	0.0	59.8	98.5	1.5	0.0

*Reaction condition: 0.5 g substrate, 5.2 mmol nickel-based alloy catalyst, 20 mL H_2O, 4 MPa H_2, 300 ℃, 5 h.

†0.3 g substrate.

Supplementary Table 4 Carbon yield and product distribution of CH_4, C2-C4 hydrocarbons and CO_2 after the reaction with different substrates and catalysts

Entry*	Substrate	Carbon yield (%)						Product distribution (mol%)		
		CH_4	C_2H_6	C_3H_8	C_4H_{10}	CO_2	Total	CH_4	C_2-C_4	CO_2
1	Methanol†	96.5	0.5	0.0	0.0	0.0	97.0	99.7	0.3	0.0
2	Methanol‡	21.7	0.1	0.0	0.0	2.9	24.7	87.9	0.3	11.8
3	Ethanol†	46.3	0.1	0.0	0.0	0.0	46.4	99.9	0.1	0.0
4	Ethanol‡	10.3	0.1	0.0	0.0	0.2	10.6	97.5	0.3	2.2

*Reaction condition: 0.5 g substrate, 20 mL H_2O, 4 MPa H_2, 250 ℃, 2 h.

†2.6 mmol nickel-based alloy catalyst.

‡3.1 mmol commercial nickel catalyst.

Supplementary Table 5 Weight percentage of C, H and O in lignocellulose substrates

Entry*	Sample	C (%)	H (%)	O (%)	Others (%)
1	Wheat straw	40.9	4.8	38.8	15.5
2	Corncob	43.9	5.2	44.1	6.8
3	Pine	40.9	6.1	39.7	13.3
4	Birch	49.8	5.4	40.1	4.7
5	Poplar	42.5	4.5	41.7	11.3
6	Beech	43.0	7.2	39.5	10.3
7	Organosolv lignin	54.9	5.3	28.5	11.3

*According to the weight percentage of C, H and O, the mole numbers of C, H and O in a certain amount of lignocellulose substrates could be calculated. Others contained N, S and ash.

Supplementary Table 6 Carbon yield and product distribution of CH_4, C2-C4 hydrocarbons and CO_2 with microcrystalline cellulose, xylan and organosolv lignin

Entry*	Substrate	Carbon yield (%)						Product distribution (mol%)		
		CH_4	C_2H_6	C_3H_8	C_4H_{10}	CO_2	Total	CH_4	C_2-C_4	CO_2
1	Microcrystalline cellulose	75.5	6.2	4.6	2.8	1.0	90.1	92.2	6.5	1.3
2	Xylan†	67.9	7.8	2.9	0.9	0.0	79.5	93.0	7.0	0.0
3	Organosolv lignin‡	27.5	0.6	0.5	0.2	0.0	28.8	98.3	1.7	0.0

*Reaction condition: 0.47 g microcrystalline cellulose, 5.2 mmol nickel-based alloy catalyst, 20 mL H_2O, 4 MPa H_2, 270 ℃, 5 h.

†0.21 g Xylan.

‡0.21 g Organosolv lignin.

Supplementary Table 7 Proximate and ultimate analysis of wood

Proximate analysis (%)		Ultimate analysis (%)	
Moisture	5.9	Ash	8.1
Fixed carbon	14.5	Carbon	43
Volatile	79.0	Hydrogen	7.2
Ash	0.6	Nitrogen	0.8
		Sulphur	1.4
		Oxygen	39.5

Supplementary Table 8 Components of key stream

	Feedstock	Hydrogen	Crude product	Recycle H_2	Natural gas
Mass (t/h)	1239.8	8039.4	1269.6	1712.6	28.1
Temperature (℃)	25	25	300	25	25
Pressure (atm)	1	5	101.65	5	5
Mass fraction					
H_2O	94.45		97.65		0.19
Wood	5.55				
Hydrogen		100.00	0.13	92.59	0.37
Methane			2.00	7.41	89.94
Ethane			0.11	0	4.86
Propane			0.07	0	3.22
Butane			0.03	0	1.42

Supplementary Table 9 Assumptions for the estimation of total product cost

Component		Base
Total capital investment		
Inside battery limits	(1.1)	Installed cost of all equipment
Installed cost of cryogenic system		"six-tenths rule" based on previous work[2]
Installed cost of other equipment		Aspen Process Economic Analyzer
Outside battery limits	(1.2)	20% of (1.1)
Indirect costs	(1.3)	60% of ((1.1) + (1.2))
Fixed capital investment	(1.4)	(1.1) + (1.2) + (1.3)
Working capital	(1.5)	5% of (1.4)
Total production cost		
Raw materials	(2.1)	Wood price: 200 CNY/t<br>Hydrogen price: 10000 CNY/t<br>Process water: 15 CNY/t
Utilities	(2.2)	Cooling water: 3 CNY/t<br>Electricity: 0.5 CNY/kW ·h
Operating & maintenance cost	(2.3)	
Operating labors	(2.3.1)	60 operators,<br>100000 CNY/operator/year
Direct supervisory & clerical labor	(2.3.2)	20% of (2.3.1)
Maintenance and repairs	(2.3.3)	2% of (1.4)
Operating supplies	(2.3.4)	0.8% of (1.4)
Laboratory charge	(2.3.5)	15% of (2.3.1)
Depreciation	(2.4)	Life period 20y, salvage value 4%
Plant overhead cost	(2.5)	60% of ((2.3.1) + (2.3.2) + (2.3.3))
Administrative cost	(2.6)	2% of product cost
Distribution and selling cost	(2.7)	2% of product cost

Supplementary Table 10 Molar ratio of consumed H_2 to generated carbon in gas phase in the experiment

Reaction path	n_{H2}/n_C
Coal to natural gas[3]	~3.0
Our work*	1.6

*In our work, the content of C, H and O in beech sawdust was 43.0%, 7.2% and 39.5%, respectively.

In the catalytic conversion of beech sawdust at 300 ℃ for 5 h, the H_2 consumption during the reaction was 5.1×10^{-2} mol, and the amount of generated carbon in gas phase after the reaction was quantified to be 3.1×10^{-2} mol. Thus, the molar ratio of consumed H_2 to generated carbon in gaseous phase was 1.6.

Supplementary References

1. Si, X. et al. A strategy for generating high-quality cellulose and lignin simultaneously from woody biomass. *Green Chem.* **19**, 4849-4857 (2017).
2. Spallina, V. et al. Techno-economic assessment of different routes for olefins production through the oxidative coupling of methane (OCM): advances in benchmark technologies. *Energy Convers. Manage.* **154**, 244-261 (2017).
3. Kopyscinski, J., Schildhauer, T. J. & Biollaz, S. M. A. Production of synthetic natural gas (SNG) from coal and dry biomass-a technology review from 1950 to 2009. *Fuel* **89**, 1763-1783 (2010).
